# Integrated versus standalone home-based records for reproductive, maternal, newborn, and child health in Nepal: A comparative qualitative study with descriptive quantitative profiling

**DOI:** 10.1371/journal.pone.0346253

**Published:** 2026-04-03

**Authors:** Sudim Sharma, Anjali Neupane, Dikshya Kandel, Pratibha Chalisay, Sabina Marasini, Budhi Setiawan, Deepak Chandra Bajracharya, Shyam Raj Upreti, Leela Khanal, Haruko Yokote, Chahana Singh, Kshitij Karki

**Affiliations:** 1 Group for Technical Assistance, Lalitpur, Nepal; 2 GTA Foundation, Lalitpur, Nepal; 3 Kathmandu University Hospital, Dhulikhel Hospital, Dhulikhel, Nepal; 4 United Nations Children's Fund, Lalitpur, Nepal; 5 Independent Researcher, Kathmandu, Nepal; National University of Sciences and Technology, PAKISTAN

## Abstract

**Background:**

Home-Based Records (HBRs) are personal health documents intended to improve continuity of care and caregiver engagement across reproductive, maternal, newborn, and child health (RMNCH) services. In Nepal, both standalone (sHBR) and integrated (iHBR) models are implemented, yet comparative evidence on their utilization and implementation challenges is limited. This study examined utilization patterns and system-level barriers associated with sHBR in Madhesh Province and iHBR in Koshi Province.

**Methods:**

We conducted a comparative qualitative study with descriptive quantitative profiling between May 17 and August 27, 2024. A total of 100 semi-structured in-depth interviews were completed with caregivers, health workers, Female Community Health Volunteers, and program managers across two provinces. The study applied “kuragraphy,” an ethnographic approach integrating interviews and field observations to construct contextual case narratives. Socio-demographic data were analyzed descriptively using the statistical package for the social Sciences (SPSS). Informed by the Human Centered Design (HCD) approach, the qualitative data were thematically analyzed in Excel using the Journey to Health and Immunization (JTHI) framework.

**Results:**

Caregivers widely perceived HBRs as essential documents, primarily for immunization tracking and future service access. The iHBR was viewed as more comprehensive and user-friendly, particularly due to its illustrations, which improved comprehension among low-literacy users. However, understanding remained limited among illiterate and marginalized populations. Family involvement in record management was minimal and largely confined to mothers. Implementation barriers included inadequate training – particularly for iHBR use, limited decision-making authority among frontline health workers, incomplete documentation of non-immunization components, poor material quality of sHBR, and concerns regarding the sustainability of donor-supported iHBR initiatives.

**Conclusion:**

HBR utilization in Nepal is shaped by caregiver literacy, gender dynamics, and health-system readiness. Strengthening training, supportive supervision, user-centered design, and sustainable supply mechanisms will be essential to optimize HBR effectiveness and support equitable RMNCH service delivery.

## Introduction

Home-based records (HBRs) are health documents that systematically capture an individual’s history of health service utilization, particularly across the reproductive, maternal, newborn, and child health (RMNCH) continuum. Maintained at the household level, HBRs complement facility-based registers and function as portable, longitudinal records that travel with the client across service points [1]. By documenting key clinical encounters and preventive services, HBRs are intended to strengthen continuity of care, facilitate communication between providers and caregivers, and promote informed decision-making at the household level. Globally, HBRs are implemented in more than 163 countries and are widely recognized as practical instruments for improving service tracking and caregiver engagement [1,2].

Despite their global adoption, HBR formats and implementation approaches vary substantially. In many settings, standalone home-based records (sHBRs), such as antenatal care (ANC), postnatal care (PNC), or immunization cards, focus on documenting specific service components [1,3]. More recently, some countries have begun piloting digital or hybrid formats to enhance durability and data integration [1,2]. Evidence indicates that well-designed HBRs can improve service uptake, caregiver knowledge, and documentation completeness [1,4–6]. However, these benefits are contingent upon contextual factors, including literacy levels, quality of counseling, health worker engagement, and the reliability of supply systems [4].

In Nepal, standalone HBRs have been embedded within the national health system since 1979, primarily in the form of immunization and maternal health cards [1,7–9]. Although these records are widely distributed, their retention and effective use remain inconsistent [10]. National data from 2016 indicate that only about half of caregivers retained child health cards, with barriers including low literacy, limited understanding of long-term value, inadequate or insensitive counseling, and poor material durability [11]. Furthermore, suboptimal documentation practices, characterized by incomplete or selective recording, have been reported across other low- and middle-income countries (LMICs), undermining the reliability of HBRs as longitudinal clinical tools [5]. Together, these challenges limit the capacity of sHBRs to function as comprehensive instruments for continuity of care.

Recognizing these limitations, the Government of Nepal introduced an integrated Home-Based Record (iHBR), known as the *“Aama Pustika”* (Mother’s Handbook), in fiscal year 2020/21 [12]. Unlike standalone cards, the iHBR was designed to consolidate RMNCH documentation within a single volume while simultaneously incorporating structured health education content. The integration of service records and educational messaging reflects an effort to address both documentation gaps and caregiver knowledge barriers. Encouraged by early implementation experience, the Government has articulated plans for a broader national scale-up [3].

However, the transition from standalone to integrated systems unfolds within a complex and evolving federal governance structure. Nepal’s decentralized health system distributes responsibilities across federal, provincial, and local governments, creating potential coordination and accountability challenges. Persistent issues – including delays in budget disbursement, procurement inefficiencies, inconsistent supervisory mechanisms, and workforce capacity constraints may affect the effective implementation and sustainability of the iHBR [13,14]. In this context, both sHBRs and iHBRs remain critical tools for navigating healthcare delivery in resource-limited settings, supporting service continuity, caregiver engagement, and the use of point-of-care data [11].

Although global evidence highlights the potential advantages of integrated records [1,4–6], comparative evidence examining how different HBR models function within the same national health system remains limited. Understanding how caregivers, frontline providers, and program managers interact with these tools and how literacy, gender dynamics, social stratification, and institutional readiness influence their use is essential for informing policy decisions regarding nationwide implementation [4–6]. Guided by the JTHI framework and informed by HCD principles [15], this study examined how sHBR in Madhesh Province and iHBR in Koshi Province are utilized and implemented within routine service contexts. The ultimate objective of this study was to generate contextually relevant evidence that can inform improvements in record design, provider capacity strengthening, supply chain reliability, and equitable distribution, thereby reinforcing RMNCH service delivery and contributing to a more responsive and data-informed health system in Nepal.

To align with this system-oriented framing, this qualitative inquiry was guided by two interrelated research questions. First, how do caregiver engagement, perceived value of the record, and safekeeping behaviors differ between iHBR and sHBR models within their respective service contexts? Second, what institutional and social mechanisms, particularly municipal supply chain management, donor dependency, and health-worker training systems, shape the sustainability and effective implementation of integrated home-based record systems? By addressing these questions, the study ensures that both demand-side and supply-side dynamics are examined in a coherent analytical lens.

## Methods and materials

### Study design and settings

This study employed an exploratory qualitative approach with a cross-sectional design, supported by descriptive quantitative profiling to contextualize participant characteristics. Research was conducted in Koshi Province across three sites implementing the iHBR system: Khandbari Municipality, Dhanpalthan Rural Municipality, and Mai Municipality. These were compared against three sites in Madhesh Province utilizing the sHBR system: Siraha Municipality, Janakpurdham Sub-Metropolitan City, and Ekdara Rural Municipality.

### Sampling

The study included a total of 100 participants, evenly distributed between the two provinces, with 50 participants selected from each. A purposive sampling strategy was used to ensure representation across the respondents. Participants were categorized into three distinct user groups, following the classification outlined in the WHO, UNICEF, and JICA HBR guide [16]. Category I – Caregivers: This group included mothers who had either lost or retained their HBR, migrant mothers, fathers, and other family members involved in caregiving. Caregivers who both retained and did not retain the child health card (referred as sHBR is this study) and iHBR were included in this category. Category II – Health Workers/Volunteers: This group comprised nurses, midwives, FCHVs, and vaccinators. Category III – Program Managers: Participants in this category included provincial and municipal-level managers responsible for Maternal and Neonatal Health (MNH) and immunization programs, as well as health facility managers.

### Data collection tools and techniques

Semi-structured interview guides were used for each participant category to ensure context-specific and role-relevant data collection. A comparative approach was adopted to explore similarities and differences in the implementation of standalone and integrated Home-Based Records (sHBR/iHBR) across the two provinces. To preserve methodological consistency and enable meaningful comparison, identical thematic domains, question structures, and data collection procedures were applied in both settings.

The tools were developed using the JTHI framework and informed by HCD principles [17]. The JTHI framework structured the interview guides around defined service “milestones,”. For instance, the Preparation Cost and effort, domain-informed specific interview questions such as, What efforts do you make to keep HBRs safe? to assess perceived document value and domestic safekeeping. During analysis, while the “Point of Service” domain served as a primary deductive category for coding counseling quality, the emergent concept of ‘disrespectful provider behavior’ was inductively identified and nested within the “Experience of Care” domain to capture relational dynamics that were not explicitly predefined in the original framework.

**Fig 1:**
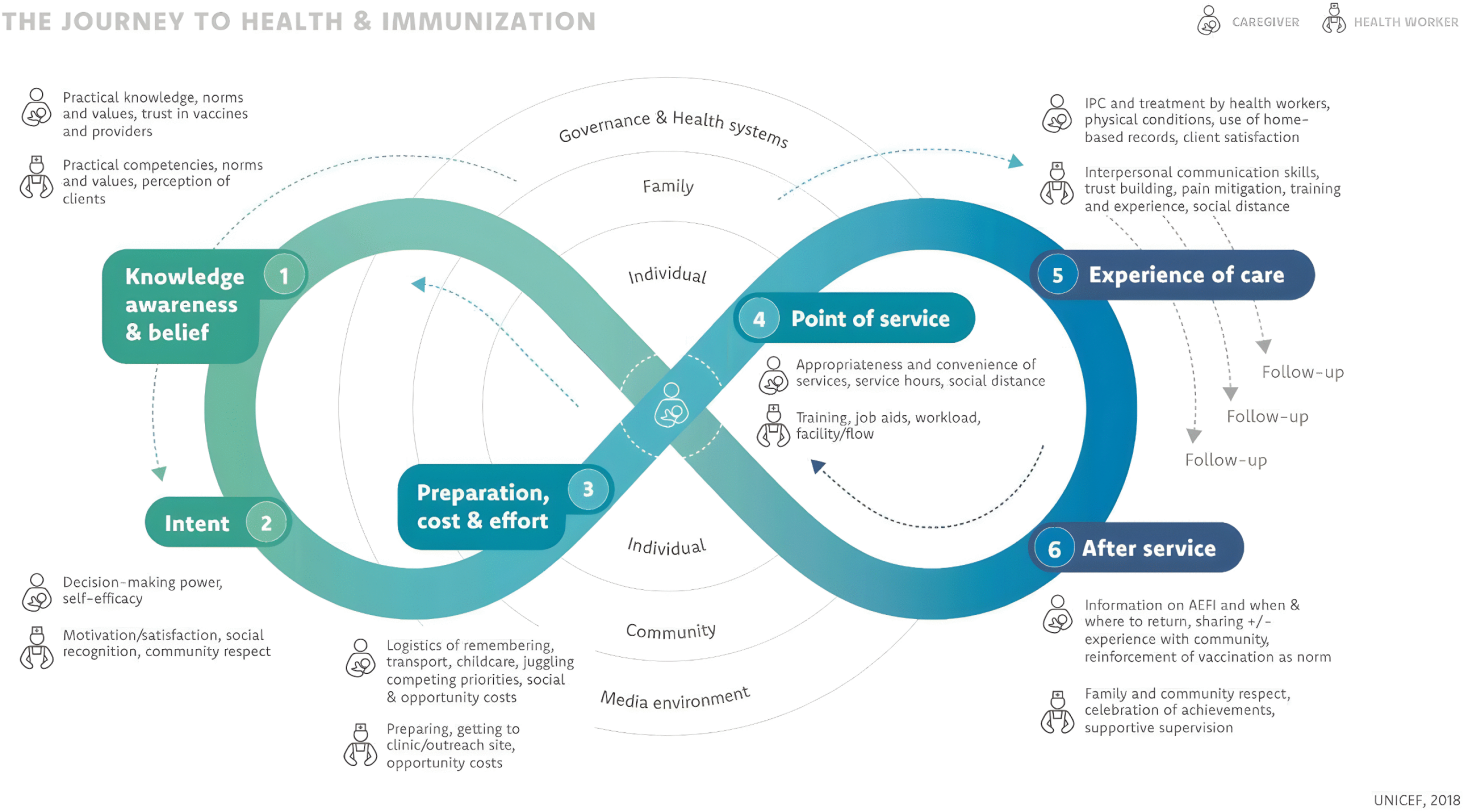
Journey to Health and Immunization (Reproduced from: Demand for Health Services: A Human-Centered Field Guide for Investigating and Responding to Challenges (BETA edition), UNICEF, p. 52 under a CC BY license, with permission from UNICEF original copyright 2018).

Conceptually, the JTHI framework delineates two interconnected journeys: [1] the caregiver’s journey shaped by social norms, financial and emotional constraints, and trust in the health system; and [2] the provider and system journey including health workers, Female Community Health Volunteers (FCHVs), and [3] program managers, shaped by training, planning processes, management structures, workload, and community engagement [18]. By examining both journeys simultaneously, the framework facilitates a systems-level understanding of how HBR-related interventions are experienced at the household level and operationalized within service delivery environments. HBR-related experiences were explored across six pre-defined JTHI domains, and qualitative data were coded and mapped accordingly (Fig 1).

The interview tools were initially drafted in English and subsequently translated into Nepali. To enhance content validity and reliability, the research team reviewed relevant systematic reviews [5,6], and South Asian studies to ensure comprehensive domain coverage [16,19]. The tools were further refined through a consultative workshop with the Technical Working Group at the Family Welfare Division (FWD) of the Ministry of Health and Population (MoHP), Nepal. This group comprised key governmental stakeholders responsible for HBR rollout, development partners (including WHO, UNICEF, and JICA), and end-user representatives.

Pre-testing was conducted in Kirtipur Municipality with ten participants representing caregivers, health workers, and FCHVs. Feedback from the pilot informed revisions to improve clarity, sequencing, and contextual relevance of questions.

Data collection techniques included in-depth interviews and structured on-site observations across the three primary user groups. The study employed “kuragraphy,” an ethnographic method that examines subjects within their natural settings and synthesizes interview data and field observations into coherent case narratives [20,21]. Unlike conventional ethnography, which typically requires prolonged immersion to understand broad cultural structures, kuragraphy was selected for its ability to rapidly yet systematically capture focused behavioral and social insights regarding specific ‘service journeys’ or ‘social scripts’ [22–24]. This approach proved particularly appropriate for analyzing the discrete interaction points of the HBR journey and the real-time social dynamics, such as provider-client trust and power imbalances, of clinical encounters. Furthermore, on-site observations provided additional insight into the domestic context of health-system interactions, contextualizing behavioral barriers through the direct study of record handling and storage processes within households and health facilities.

To ensure uniformity in the application of these techniques, field researchers fluent in both Nepali and English received standardized training in focused interviewing and observational recording prior to the study. Data collection was conducted between May 17 and August 27, 2024, during which all interviews were audio-recorded with explicit participant consent. Throughout this period, researchers maintained detailed field notes to capture the contextual, behavioral, and interactional dynamics of each encounter. Following the data collection, all audio recordings were transcribed verbatim and translated into English for final analysis.

### Data analysis

Socio-demographic characteristics were summarized using descriptive statistics in SPSS to provide a contextual baseline for the thematic analysis. Qualitative data were analyzed using a systematic thematic analysis conducted in Microsoft Excel. An initial codebook was developed based on empirical data and subsequently mapped to the JTHI framework through deductive coding. Two independent coders applied the coding framework, achieving an intercoder agreement of 93%. Discrepancies and newly emerging codes were resolved through iterative discussion and consensus.

### Ethical considerations

This study obtained ethical approval from the Nepal Health Research Council (NHRC) (Ref. No.: 2005, Registration No.: 203_2024). All the participants were informed about the study’s objectives and purpose, and written informed consent was obtained from each participant. Additional consent was obtained to record the interviews. Participants’ safety and confidentiality were maintained throughout the study.

## Results

### Sociodemographic information

Table 1 presents the sociodemographic profile of the 78 participants from Madhesh and Koshi provinces across Categories I and II, showing that most participants were women, aged 20–39 years, predominantly Hindu (87.2%), married (85.9%), and living in joint or extended families (69.2%). Overall, participants were largely from Janajati (35.9%), Dalit (20.5%), Madhesi (21.8%), and Brahmin/Chhetri (20.5%) groups, with secondary-level education (37.2%), civil service employment (37.2%), and monthly incomes of NPR 50,000 or above (37.2%) being common **Table 1**.

**Table 1:** Sociodemographic characteristics of Categories I and II participants (n = 78).

	Madhesh n (%)		Koshi n (%)		
Category	*Cat. I* *(n = 21)*	*Cat. II* *(n = 18)*	*Cat. I* *(n = 21)*	*Cat. II* *(n = 18)*	Total n (%)
**Age**					
< 20	2 (9.5)	–	1 (4.8)	–	3 (3.84)
20-39	15 (71.4)	11 (61.1)	15 (71.4)	11 (61.1)	52 (66.67)
40-59	3 (14.3)	6 (33.3)	2 (9.5)	6 (33.3)	17 (21.79)
≥ 60	1 (4.8)	1 (5.6)	3 (14.3)	1 (5.6)	6 (7.6923)
**Sex**					
Male	7 (33.33)	4 (22.2)	9 (42.9)	1 (5.6)	21 (26.923)
Female	14 (66.7)	14 (77.8)	12 (57.1)	17 (94.4)	57 (73.07)
**Ethnicity**					
Brahmin/Chhetri	5 (23.8)	3 (16.7)	1 (4.8)	7 (38.9)	16 (20.51)
Janajati	3 (14.3)	6 (33.3)	13 (61.9)	6 (33.3)	28 (35.89)
Dalit	8 (38.1)	2 (11.1)	5 (23.8)	1 (5.6)	16 (20.51)
Madhesi	5 (23.8)	6 (33.3)	2 (9.5)	4 (22.2)	17 (21.79)
Muslim	–	1 (5.6)	–	–	1 (1.28)
**Religion**					
Hindu	21 (100)	17 (94.4)	15 (71.4)	15 (83.3)	68 (87.19)
Buddhism	–	–	5 (23.8)	–	5 (6.41)
Islam	1 (5.6)	1 (5.6)	–	–	2 (2.56)
Kirat	–	–	1 (4.8)	–	1 (1.28)
**Family type**					
Nuclear	5 (23.8)	6 (33.3)	3 (14.3)	10(55.6)	24 (30.76)
Joint/Extended	16 (76.2)	12 (66.7)	18 (85.7)	8 (44.4)	54 (69.23)
**Number of children**					
No children	1 (4.8)	–	2 (9.5)	4 (22.2)	7 (8.97)
1-2	11 (52.4)	11 (61.1)	13 (61.9)	12 (66.7)	47 (60.25)
3-4	6 (28.6)	7 (38.9)	4 (19)	2 (11.1)	19 (24.35)
5 and above	3 (14.3)	–	2 (9.5)	–	5 (6.41)
**Literacy**					
Illiterate	1 (4.8)	–	4 (19)	–	5 (6.41)
Literate (no formal education)	9 (42.9)	1 (5.6)	4 (19)	–	14 (17.94)
Basic primary (Class 1–8)	3 (14.3)	3 (16.7)	5 (23.8)	6 (33.3)	17 (21.79)
Secondary (Class 9–12)	7 (33.3)	9 (50)	6 (28.6)	7 (38.9)	29 (37.17)
Higher level (Bachelor+)	1 (4.8)	5 (27.8)	2 (9.5)	2 (11.1)	10 (12.82)
**Marital status**					
Married	19 (90.5)	16 (88.9)	17 (81)	15 (83.3)	67 (85.89)
Unmarried	1 (4.8)	1 (5.6)	3 (14.3)	3 (16.7)	8 (10.25)
Widow	1 (4.8)	1 (5.6)	–	–	2 (2.56)
**Employment**					
Agriculture	5 (23.8)	2 (11.1)	6 (28.6)	3 (16.7)	16 (20.51)
Labor	1 (4.8)	–	–	–	1 (1.28)
Civil Service	2 (9.5)	12 (66.7)	3 (14.3)	12 (66.7)	29 (37.17)
Company/Business	1 (4.8)	1 (5.6)	5 (23.8)	1 (5.6)	8 (10.25)
Unemployed	1 (4.8)	–	1 (4.8)	–	2 (2.56)
Retired	2 (9.5)	–	1 (4.8)	–	3 (3.84)
Housewife	9 (42.9)	3 (16.7)	5 (23.8)	2 (11.1)	19 (23.35)
**Monthly income**					
<10000	2 (9.5)	–	1 (4.8)	1 (5.6)	4 (5.12)
10000-20000	7 (33.3)	–	2 (9.5)	2 (11.1)	11 (14.1)
20000-30000	3 (14.3)	4 (22.2)	6 (28.6)	1 (5.6)	14 (17.94)
30000-40000	4 (19)	3 (16.7)	5 (23.8)	1 (5.6)	13 (16.66)
40000-50000	1 (4.8)	–	4 (19)	2 (11.1)	7 (8.97)
≥ 50000	4 (19)	11 (61.1)	3 (14.3)	11 (61.1)	29 (37.18)

Table 2 summarizes the sociodemographic characteristics of Category III participants (program managers) from Madhesh and Koshi provinces (n = 22). Most participants were aged 20–39 years (50.0%), male (59.1%), and had at least a bachelor’s level of education (59.1%), reflecting a relatively young and well-educated managerial workforce involved in MNH, immunization, and health facility management Table 2.

**Table 2:** Sociodemographic characteristics of category III participants (n = 22).

Category	Madhesh n (%)	Koshi n (%)	Total n (%)
**Age**			
< 20	1 (9.09)	–	1 (4.55)
20-39	5 (45.45)	6 (54.54)	11 (50)
40-59	4 (36.36)	4 (36.36)	8 (36.36)
≥ 60	1 (9.09)	1 (9.09)	2 (9.09)
**Sex**			
Male	6 (54.5)	7 (63.6)	13 (59.1)
Female	5 (45.5)	4 (36.4)	9 (40.9)
**Literacy**			
+2/Diploma	4 (36.4)	5 (45.5)	9 (40.9)
Bachelor’s degree	5 (45.5)	4 (36.4)	9 (40.9)
Master’s degree	2 (18.2)	2 (18.2)	4 (18.18)

### Case narrative excerpts

#### Excerpt 1: Household dynamics and record loss.

Aarti (name changed), a mother from Koshi province, managing her household alone while her husband worked abroad, initially valued the iHBR as a vital resource during her newborn’s high-risk hospitalization. However, the record was eventually lost after her child used the colorful booklet as a toy while Aarti was preoccupied with domestic chores. Despite recognizing its importance, she did not report the loss to health workers, citing the overwhelming burden of her daily household responsibilities.

#### Excerpt 2: User empowerment and retention.

Urmila (name changed), a mother from the same province, despite only having a basic education, was motivated by health workers to read the iHBR’s embedded educational messages. She described the pictorial guidance as “empowering” and subsequently carried the record to every visit, ensuring complete documentation across ANC, PNC, and growth monitoring sections. Her experience suggests that comprehensive, user-centered design can bridge literacy gaps and foster proactive record-keeping. (*Full narratives for both cases are provided in* S1 File)

### Theme 1: Knowledge, awareness, and belief

Knowledge and awareness regarding HBR utility revealed a significant provincial divide. In Koshi Province, caregivers demonstrated a holistic understanding of the iHBR, viewing it as a dual-purpose clinical and educational tool. Conversely, caregivers in Madhesh Province predominantly held a narrow, functional view of the sHBR, often seeing it solely as a “vaccination card” required for service access.

*“iHBR is important for tracking service records, receiving service, getting information on care during pregnancy and after childbirth, and it facilitates a child’s school admission and going abroad”.*

*“If I do not carry child health card while visiting the health facility (HF) for immunization, the health worker will not vaccinate my child”.*

Despite these differences, a universal lack of awareness regarding replacement procedures for lost records persisted in both regions. In Koshi, one mother remarked,

*“I lost my child's health card. But I did not know we could get a new one from a health facility. I was confused and did not follow up with the HF to get a new one”.* In Madhesh, the belief that the record had no purpose after vaccinations were complete led to disposal:

*“The sHBR is of no use once the child is grown up. So, we no longer have it”.*

### Theme 2: Intent

The study identified a clear contrast in proactive engagement and decision-making power between the two provinces. Intent on safeguarding the record was notably higher in Koshi, driven by the belief that the iHBR was a vital resource for long-term health milestones, such as “nutritional care.” A mother in Koshi noted,

*“My child is growing up, and I find iHBR important for his nutritional care”.*

In Madhesh, maternal autonomy regarding health-seeking and document management was frequently constrained by joint family dynamics. As one mother from Madhesh explained:

*“My husband and in-laws take the decision when it comes to seeking RMNCH services”.*

Fathers in Koshi demonstrated higher levels of support and awareness compared to their counterparts in Madhesh. One father from Koshi shared,

*“I remind my wife to take the iHBR during the HF visit. When possible, I accompany wife to receive RMNCH services”.*

In sharp contrast, a father in Madhesh admitted to a lack of oversight and involvement in record maintenance:

*“I have not paid attention to keeping the sHBR safely. It must have been lost”.*

On the supply side, health workers in both provinces felt detached from strategic HBR management and policy discussions. This lack of involvement in the decision-making process reportedly led to decreased motivation and professional commitment to the program. A nurse in Madhesh reflected this sentiment:

*“My limited role in decision-making has, at times, affected my motivation for active engagement.”*

Conversely, health workers who possessed some level of authority, particularly in Koshi, reported that being “involved in stock management, record keeping, and verification of HBR” increased their determination to improve RMNCH services.

### Theme 3: Preparation, cost, and effort

Structural and logistical challenges varied significantly across the study sites, with Koshi Province facing critical sustainability risks following a donor exit, while Madhesh Province struggled with chronic stock-outs and the poor material durability of the standalone records. Caregivers in Madhesh frequently described the extreme measures required to preserve their documents due to inferior paper quality. As one mother in Madhesh recounted:

*“I had kept the sHBR in a plastic bag, but rainwater leaked into the house and damaged it. If it had been laminated, it would have been safe”.* These environmental vulnerabilities often led to the loss or misplacement of essential health data.

In Koshi, the discontinuation of the donor-supported pilot created widespread confusion regarding the future of the integrated model. A nurse in Koshi highlighted the resulting inequity in access:

*“Only* *a limited number of mothers got access to iHBR. As iHBR is stocked out, we have been giving sHBR to users”*.

While most municipalities struggled with these transitions, Dhanpalthan Rural Municipality successfully demonstrated local ownership by independently funding the printing and distribution of iHBRs to maintain service continuity.

Supply mismatches remained a pervasive issue in Madhesh, where provincial distribution often failed to align with actual facility needs. One vaccinator noted:

*“There have been many incidents of stock out of sHBR this year. Province level does not distribute sHBR based on need”.*

Beyond supply chains, health workers in both provinces identified human resource constraints and a lack of physical infrastructure, such as dedicated cupboards, as major barriers to effective record management. For instance, a nurse in Koshi noted,

*“We do not have enough staff to provide the services, so we cannot manage the logistics properly,”* while a vaccinator in Madhesh added,

*“There is not enough space to manage the logistics properly. We do not have adequate space for even service delivery”.*

### Theme 4: Point of service

While satisfaction with health facility availability was generally high across both provinces, significant systemic barriers such as chronic staff shortages and facility overcrowding hindered the quality of clinical counseling and record management. Most mothers received their HBR during their first RMNCH visit, yet the delivery of care was often compromised by high patient volume.

Caregivers frequently reported “long waiting times due to overcrowding” when receiving services. One migrant mother in Koshi noted:

*“The health facility has a high flow of patients, sometimes I have to wait in line for longer hours to receive RMNCH services”.*

These delays often shortened the time available for health workers to provide meaningful counseling on the record's contents.

Providers specifically highlighted those structural limitations, such as “the lack of a separate or safer space” for ANC check-ups, caused notable hesitancy among pregnant women. A midwife in Madhesh shared:

*“There is no separate room for ANC check-up. Sometimes, this causes hesitancy among pregnant women for ANC check-up.”*

Workload pressures further exacerbated these challenges, particularly in smaller facilities. As one vaccinator in Koshi explained:

*“We only have 3 staff at the HF. When one is absent, our workload subsequently increases, creating difficulties in RMNCH service provision and counseling on HBR”.*

Additionally, the absence of dedicated monitoring mechanisms meant that there was little institutional support to ensure complete recording or effective implementation of the HBR system during these busy periods.

### Theme 5: Experience of care

A stark difference was observed in how records were handled during provider-client interactions, revealing a significant “gatekeeping” disparity between the two provinces. In Koshi, health workers leveraged the iHBR's visual design as an essential counseling aid to bridge communication gaps, particularly with caregivers of lower literacy. As one vaccinator in Koshi noted:

*“As iHBR is pictorial, it is easy for us to counsel service users referring to iHBR”.*

In contrast, service access in Madhesh was often strictly contingent on the physical presentation of the standalone card, which functioned as a barrier to care for those who failed to bring it. One mother from the marginalized community in Madhesh emphasized the pressure of this requirement:

*“We have to remember to carry the sHBR while visiting HF, otherwise they do not vaccinate the child”.*

Conversely, providers in Koshi demonstrated greater flexibility and a commitment to service continuity by utilizing facility registers when records were missing. A midwife in Koshi explained:

*“If the service users forget their HBR, I refer to the service register, update their records, and provide a service note... We do not send them back”.*

Despite these divergent approaches to service access, direct observations across both regions highlighted persistent documentation challenges. Even in instances where immunization records were filled completely, significant gaps were found in sections dedicated to growth monitoring, Vitamin A supplementation, and exclusive breastfeeding (EBF).

### Theme 6: After service

Reflections on the records after service underscored a strong user preference for consolidated, paper-based tools over fragmented or digital ones. In Madhesh Province, the use of multiple separate forms for tracking various RMNCH services created significant logistical confusion and increased the risk of document loss. A relative in Madhesh highlighted this burden:

*“There are different home-based records and high chances of losing them. Compiling HBR into one would make it easy to keep them safely”.*

In contrast, the integrated format in Koshi was highly valued not just for documentation, but as a longitudinal health guide. A migrant mother in Koshi shared her excitement for the tool's user-centered design:

*“When I received iHBR for the first time, I was drawn to its colorful design and illustrations... The contents in iHBR were very helpful throughout my pregnancy, postpartum period, and child's care”.*

However, the transition to this new format presented practical hurdles; health workers reported that the specific material of the iHBR made it “difficult to write in... with commonly available pens,” which could make the recording process “tedious” during peak facility hours.

Regarding the medium of the record, both study groups expressed a firm preference for physical records over digital health applications, citing significant barriers related to mobile phone access and technical literacy among caregivers. A nurse in Madhesh summarized this institutional and social reality:

*“I prefer paper-based HBR because not all mothers may have access to personalized mobile phones. Digital HBR may be appropriate for HWs but not for caregivers”.*

Finally, the “After Service” phase was often reinforced by household dynamics; caregivers who communicated with their spouses about the record were frequently reminded by family members to carry the HBR to subsequent visits, highlighting the record's role in broader family-centered health accountability.

Thematic summaries are succinctly presented below, providing a comprehensive overview of the key findings from the HBR study Table 3.

**Table 3:** Comparative thematic summaries of integrated and standalone home-based records across provinces.

Theme	Koshi Province – Integrated HBR (iHBR)	Madhesh Province – Standalone HBR (sHBR)
**1. Knowledge, Awareness & Belief**	Broad, holistic understanding of iHBR as a clinical, educational, life-course document (pregnancy, nutrition, school admission, travel). Viewed as a long-term asset. Strong perceived value beyond immunization; supports continuity of care. Common gap: No awareness of replacement procedures when lost.	Narrow functional perception as a “vaccination card” required for service access. Often discarded after immunization completion. Instrumental value only; limited awareness of broader RMNCH functions. Common gap: No awareness of replacement procedures when lost.
**2. Intent**	High safeguarding intent. Mothers demonstrate proactive engagement; fathers show supportive involvement. Record linked to future milestones (nutrition, growth, development). Health workers with logistical authority report higher motivation and ownership.	Passive retention. Maternal autonomy is constrained by joint family structures; fathers are minimally engaged in record management. Record perceived as transactional requirement for vaccination. Health workers report limited role in decision-making resulting in reduced motivation and accountability.
**3. Preparation, Cost & Effort**	Donor-supported pilot ensured initial availability; post-donor exit created sustainability uncertainty. Some municipal ownership (e.g., independent local printing) demonstrates sustainability potential. HR shortages and storage limitations affect logistics.	Chronic stock-outs due to provincial supply mismatches; distribution not need-based. Poor material durability; caregivers adopt protective measures (plastic wrapping) due to low-quality paper. HR shortages, inadequate infrastructure (no cupboards, limited space) significantly hinder record management.
**4. Point of Service**	High client load but flexible service delivery. Providers use facility registers if HBR forgotten which preserved continuity. Limited formal training yet relatively effective counseling using pictorial design. Staff shortages reduce time for detailed counseling.	High overcrowding; service frequently contingent on presenting sHBR introduce gatekeeping barrier. Training reported, but counseling quality inconsistent; structural constraints reduce meaningful engagement. Staff shortages, lack of private ANC space discourage care-seeking.
**5. Experience of Care**	iHBR used as a visual counseling aid; strengthens communication, especially with low-literacy caregivers. Greater flexibility and service ethos (“……….do not send them back”). Documentation gaps persist (growth monitoring, Vitamin A, EBF sections incomplete).	sHBR functions as compliance tool rather than counseling aid. Denial of vaccination without card reported. Similar documentation gaps in non-immunization sections.
**6. After Service**	Strong user preference for consolidated paper-based format; valued as longitudinal family health guide. Household reinforcement (spousal reminders) strengthens retention. Writing challenges due to paper material noted by providers. Both groups prefer paper over digital due to access and literacy constraints.	Fragmented multiple forms increase risk of loss and confusion; demand for consolidation. Weaker household engagement; higher probability of misplacement or disposal. Paper fragility and environmental vulnerability (rain damage) common. Both groups prefer paper over digital due to access and literacy constraints.

## Discussion

This study examined utilization patterns and implementation challenges of sHBR in Madhesh Province and iHBR in Koshi Province, highlighting both functional strengths and structural inequities that shape their use. Across both provinces, awareness of HBRs was high, and caregivers consistently recognized them as important documents not only for immunization tracking but also for school enrollment and civil identification. This multi-purpose value aligns with global evidence that HBRs often function as hybrid health–administrative tools, extending beyond clinical documentation to support broader social needs [6,25,26].

In Koshi Province, caregivers perceived the iHBR as a comprehensive health education resource. Its integration of maternal, newborn, and child health information reflects international experiences where consolidated records reinforce healthy behaviors during pregnancy and the postpartum period [1,5,6,25–27]. However, comprehension gaps persisted among marginalized and low-literacy populations. Evidence from Nigeria demonstrates that limited maternal health literacy is directly associated with lower immunization uptake, underscoring the importance of aligning record design with user literacy levels [28–30]. These findings reinforce the principle that HBR effectiveness depends not only on content inclusion but also on cognitive accessibility [27].

Utilization patterns were strongly shaped by sociodemographic characteristics. The study identified a “literacy paradox”: although 37.2% of participants had secondary education, nearly one-quarter (24.3%) were illiterate or had no formal schooling. In Madhesh, lower literacy levels were associated with a narrow perception of the sHBR as merely a vaccination card, often leading to disposal after completion of the immunization schedule. Conversely, in Koshi, the iHBR’s pictorial and educational layout broadened its perceived value, even among caregivers with limited formal education. These findings suggest that human-centered design can partially mitigate literacy-related barriers and expand functional utilization [31].

Although caregivers expressed willingness to retain HBRs for scheduled health visits, their understanding of their long-term value was often incomplete. Responsibility for safekeeping rested almost exclusively with mothers, reflecting entrenched gender norms. Similar gendered patterns have been documented in Bangladesh and Indonesia [32,33]. Given evidence that male engagement improves both HBR retention and immunization completion, the minimal involvement of fathers in Nepal represents a missed opportunity to strengthen family-centered accountability for child health documentation [34,35].

Training disparities between provinces further influenced record utilization. While most health workers in Madhesh had received orientation on the sHBR, many in Koshi reported insufficient training on the comprehensive use of the iHBR. As a result, documentation frequently focused on immunization sections, with limited attention to growth monitoring, nutrition, or developmental components. Comparable training gaps have been identified in Angola, where insufficient provider preparation constrained effective HBR implementation [36]. These findings suggest that introducing an integrated tool without structured capacity-building risks narrowing its functional scope in practice. Continuous in-service training, supportive supervision, and integration of HBR use into routine clinical workflows are essential to maximize its intended benefits.

Meaningful engagement of frontline health workers in HBR planning and design is known to enhance usability and ownership [25]. However, participants in this study reported limited involvement in strategic decision-making, with policy and design decisions centralized at higher administrative levels. A similar disconnect has been documented in Uganda, where health workers were primarily tasked with distribution rather than design or policy input [37]. In contrast, systems that delegate decision-making authority to implementers tend to demonstrate stronger accountability and sustained commitment. Institutionalizing participatory design processes may therefore strengthen long-term implementation fidelity [38–40].

Communication dynamics also differed between provinces. Health workers in Madhesh described challenges persuading caregivers to retain sHBRs, particularly when caregivers perceived the record as relevant only until immunization completion. Similar resistance has been reported in Cambodia [41]. In Koshi, however, providers highlighted the iHBR’s illustrations as effective counseling aids, particularly for caregivers with limited literacy. Pictorial design functioned as a practical communication bridge, enhancing message clarity and engagement. Studies from Cambodia, Angola, and Bangladesh similarly demonstrate that visual content improves HBR usability among low-literacy populations [36,41,42].

Despite provider claims of complete documentation, field observations revealed substantial gaps in growth monitoring, vitamin A supplementation, and EBF sections. Such selective recording undermines the holistic intent of HBR systems and has been documented in Kenya and other low- and middle-income countries [36,43]. This discrepancy between perceived and actual documentation quality indicates the need for periodic audit mechanisms and supervision focused not only on service delivery but also on record completeness.

Family structure further influenced maternal autonomy. With 69.2% of participants living in joint or extended households, decisions regarding RMNCH service utilization frequently rested with husbands or senior family members. Although women bore primary responsibility for HBR safekeeping, they did not consistently control health-related decision-making. This structural imbalance limits the transformative potential of HBRs as empowerment tools unless male partners and influential family members are systematically engaged [44–46].

Ethnic disparities also shaped the “experience of care.” Participants from Janajati (35.9%) and Dalit (20.5%) communities were more likely to report rushed or disrespectful encounters in overcrowded facilities. Health workers acknowledged perceptions that marginalized groups were less likely to follow counseling recommendations. These dynamics suggest that HBR effectiveness is mediated not only by design or training but also by relational trust and systemic bias within service delivery environments. Without respectful provider–client interactions, even well-designed records may fail to achieve their intended impact [47–49]. Material durability emerged as an operational concern. Many sHBR users criticized the poor paper quality, yet caregivers demonstrated strong commitment to retention by storing records in plastic bags or secure household containers, an approach also documented in WHO guidance [1]. Although both caregivers and providers expressed preference for the iHBR format, its reliance on donor funding raises sustainability concerns common in low-resource settings [1,26,27]. Encouragingly, one municipality in Koshi independently sustained iHBR implementation after external support ended, illustrating that local ownership can mitigate funding volatility.

Collectively, these findings indicate that expanding the iHBR model could enhance service utilization, improve documentation quality, and strengthen continuity of care. However, scale-up should be accompanied by context-sensitive design adaptation, structured workforce training, participatory governance mechanisms, and financing strategies that ensure long-term sustainability [1,5,41].

This study advances HBR theory by shifting the focus from the record as a static clinical document to a mediator of psychological ownership [1,27,50,51]. While existing theory suggests HBRs improve continuity of care [1], our findings demonstrate a “Recognition-Value Loop” [52]: the comprehensive, pictorial nature of the iHBR in Koshi transformed the record from a clinical requirement into a “Mother's Handbook” *(Aama Pustika).* This conceptual shift is critical; when a record is perceived as a life-stage resource rather than a facility-owned tool, caregivers develop a higher proactive intent for safekeeping, effectively bridging the “Literacy Paradox.” Consequently, HBR theory must now account for visual agency as a primary driver of record retention in low-literacy settings [1,17,50,53]. The application of the JTHI framework in this study offers a critical refinement of its original scope. Traditionally, the JTHI focuses on the behavioral and social drivers of the caregiver's journey [15,54]. However, our findings extend the framework by demonstrating that the caregiver’s journey is fundamentally hostage to the “Institutional Journey.”

We refined the JTHI by identifying Institutional Readiness (specifically donor-dependency and municipal supply chains) as a structural milestone that precedes the caregiver's “Point of Service.” When the institutional journey fails (e.g., stock-outs in Madhesh or discontinuation in Koshi), the caregiver’s “Intent” and “Preparation” are rendered irrelevant. Therefore, we propose an Integrated JTHI model where institutional reliability is not a background variable but an active determinant that dictates the success of individual behavioral drivers [54–60].

Several limitations warrant consideration. Beyond the cross-sectional nature of the data, the study’s theoretical approach introduced specific analytical trade-offs. While the JTHI framework provided a robust and systematic structure for the inquiry, the primary reliance on deductive coding based on its pre-defined “milestones” may have introduced framework-induced analytical constraints. This structural focus potentially suppressed peripheral emergent themes or localized social scripts that did not align directly with the framework’s caregiver or provider journeys. Geographic scope was limited, and purposive sampling constrains generalizability. Although the intercoder agreement was high at 93%, this percentage agreement does not adjust for chance agreement, which may slightly overstate the uniformity of the thematic application. Future research employing longitudinal and mixed-method designs across diverse ecological settings would provide stronger causal inference and implementation insight.

## Conclusion

This study provides the first systematic comparison of iHBR versus sHBR health record implementation in Nepal, utilizing the JTHI framework to evaluate how local governance and user engagement influence maternal and child health outcomes. The findings reveal that the iHBR model generates higher user satisfaction and improved service utilization by consolidating RMNCH information into a single, visually engaging, and educational tool. However, the study underscores that the success of such interventions is fundamentally “hostage” to the institutional journey; fragmented implementation, heavy donor dependency, and limited local decision-making authority significantly undermine the sustainability and effectiveness of the iHBR. To maximize the impact of a national scale-up, the government must move beyond treating HBRs as passive records and instead redefine them as active communication tools through structured workforce training and robust municipal-level supply forecasting. Furthermore, achieving equitable implementation requires addressing entrenched gender and literacy barriers by systematically engaging fathers and other household decision-makers in the care journey. Ultimately, these insights demonstrate that when decentralization is paired with local ownership and institutional accountability, integrated records can serve as transformative components of a data-informed health system.

## Supporting information

S1 File Case narratives. (DOCX)
