# Supplementary material for: Integrated versus standalone home-based records for reproductive, maternal, newborn, and child health in Nepal: A comparative qualitative study with descriptive quantitative profiling
Source: PLoS One. 2026 Apr 3;21(4):e0346253. doi: 10.1371/journal.pone.0346253 (PMC13048390; doi:10.1371/journal.pone.0346253)
Supplement: S1 File — (DOCX) [file pone.0346253.s001.docx]

## **Case narrative: story of a mother who lost iHBR from Mai municipality, Koshi province**

Mrs. Aarti Tamang (name changed), a 35-year-old mother of four from Mai Municipality, manages her household alone while her husband works abroad. After giving birth to three daughters, she conceived her fourth child, hoping for a son, and eventually delivered a baby boy. During her ANC visits, she received an iHBR and was counselled by health workers on its importance for tracking maternal and child health services.

Aarti's pregnancy was high-risk, and her newborn was later diagnosed with pneumonia, requiring prolonged hospitalization. During this challenging period, the iHBR became a vital resource, helping her follow medical guidance and monitor her child’s care. She described the booklet as informative, accessible, and relevant to her needs.

To ensure the safety of iHBR, she placed it in a cupboard. However, its colorful design caught the attention of her five-year-old daughter, who began using it as a toy. Preoccupied with household chores, Aarti did not intervene. Weeks later, during a routine home visit, a nurse requested the iHBR to verify immunization records, but Aarti could not locate it. Although she recognized its importance, she did not report the loss, citing her busy routine and household responsibilities.

## Case narrative: story of a mother who retains iHBR from Mai Municipality, Koshi province

Mrs. Urmila Magar (name changed), a 31-year-old mother of three, relocated to Mai Municipality two years ago. With her husband working abroad and her eldest daughter studying in Birtamod, Urmila manages the household on her own. Though she married young and has only completed basic education, she is deeply committed to securing a better future for her daughters.

Urmila was given the iHBR at Mai Hospital during the ANC visit for her youngest child, who is currently two and a half years old. Health workers counseled her on its importance and encouraged her to read the embedded health education messages. Motivated by this interaction, she read the entire booklet the same night. She found the content highly informative, practical, and empowering, providing clear guidance on maternal self-care, newborn care, nutrition, and danger signs.

Since then, Urmila has consistently carried the iHBR to all health facility visits. Her record is well-maintained, with complete documentation in key sections, including ANC, PNC, immunization, growth monitoring, and follow-up. Even today, she refers to the iHBR as a guide for her child’s nutrition and developmental milestones.

As the distribution of iHBR has been discontinued in the municipality, other mothers using sHBR often ask her how she obtained such a comprehensive tool. Urmila strongly believes that every pregnant woman should receive an iHBR and expresses hope that her daughters will have access to it during their pregnancies.
